# Chemotherapy-induced peripheral neuropathy models constructed from human induced pluripotent stem cells and directly converted cells: a systematic review

**DOI:** 10.1097/j.pain.0000000000003193

**Published:** 2024-02-21

**Authors:** Pascal S.H. Smulders, Kim Heikamp, Jeroen Hermanides, Markus W. Hollmann, Werner ten Hoope, Nina C. Weber

**Affiliations:** a Department of Anesthesiology, Amsterdam UMC location University of Amsterdam, Laboratory for Experimental Intensive Care and Anesthesiology (L.E.I.C.A.), Amsterdam, the Netherlands; b Department of Anesthesiology, Rijnstate Hospital, Arnhem, the Netherlands

**Keywords:** Chemotherapy-induced peripheral neuropathy, Cancer, Chemotherapy, Induced pluripotent stem cell, Dorsal root ganglion neuron

## Abstract

Developments in human cellular reprogramming now allow for the generation of human neurons for in vitro disease modelling. This technique has since been used for chemotherapy-induced peripheral neuropathy (CIPN) research, resulting in the description of numerous CIPN models constructed from human neurons. This systematic review provides a critical analysis of available models and their methodological considerations (ie, used cell type and source, CIPN induction strategy, and validation method) for prospective researchers aiming to incorporate human in vitro models of CIPN in their research. The search strategy was developed with assistance from a clinical librarian and conducted in MEDLINE (PubMed) and Embase (Ovid) on September 26, 2023. Twenty-six peer-reviewed experimental studies presenting original data about human reprogrammed nonmotor neuron cell culture systems and relevant market available chemotherapeutics drugs were included. Virtually, all recent reports modeled CIPN using nociceptive dorsal root ganglion neurons. Drugs known to cause the highest incidence of CIPN were most used. Furthermore, treatment effects were almost exclusively validated by the acute effects of chemotherapeutics on neurite dynamics and cytotoxicity parameters, enabling the extrapolation of the half-maximal inhibitory concentration for the 4 most used chemotherapeutics. Overall, substantial heterogeneity was observed in the way studies applied chemotherapy and reported their findings. We therefore propose 6 suggestions to improve the clinical relevance and appropriateness of human cellular reprogramming–derived CIPN models.

## Abstract 2

Supplemental Digital Content is Available in the Text.

## 1. Introduction

The global incidence of cancer was estimated at 17 million in 2018 and is expected to further increase to 26 million by 2040.^64^ A large proportion of these patients will be treated with a regimen that consists of chemotherapy, among other treatment modalities. Yet, although the anticancer effects of chemotherapeutic treatment are undisputed, its side effects and sequelae can have a substantial impact on the quality of life of patients.^28^

Chemotherapy-induced peripheral neuropathy (CIPN) is one of the most common long-term adverse effects of chemotherapy, occurring in up to 85% of patients, depending on the administered chemotherapeutic agent and dose.^68^ Chemotherapy-induced peripheral neuropathy is characterized as a predominantly sensory neuropathy, manifesting as changes in sensation and (severe) neuropathic pain.^58,68^ Therefore, CIPN regularly leads to dose reduction or discontinuation of antineoplastic therapy. Although urgently needed, preventive strategies and treatment options for CIPN are currently limited.^13,58,68^ This is particularly troubling considering the expected increase in prevalence of CIPN, caused by improved cancer survival rates.^58^

Laboratory studies investigating CIPN have historically been conducted using animal models, in part because of the inaccessibility of the human (peripheral) nervous system for research purposes.^11,13,38^ Although animal studies have provided valuable insights concerning CIPN, results have been difficult to translate to the human setting.^30,38^ This is particularly relevant to studies investigating potential analgesic treatment options because considerable genetic differences exist between humans and other species regarding the nociceptive system. For example, the expression levels and electrophysiological characteristics of prominent nociceptive markers differ substantially between small rodents and humans.^16,46^

Meanwhile, progress in stem cell biology has led to the development of reprogramming and differentiation protocols that can be used to produce human nociceptive dorsal root ganglion (DRG) neurons from induced pluripotent stem cells (iPSCs), embryonic stem cells (ESCs), and directly converted cells (DCCs).^29,53^ These techniques have since been adopted to model CIPN, resulting in the description of numerous laboratory models constructed from human neurons. Ultimately, these in vitro systems may be applied to investigate CIPN's pathophysiological mechanisms and explore pharmacological treatment strategies.^11^ However, published model descriptions vary widely in cellular setup and method of CIPN induction. This systematic review therefore provides a critical analysis of available models and their methodological considerations (ie, used cell type and source, CIPN induction strategy and validation method) for researchers willing to incorporate human in vitro models of CIPN in their research.

## 2. Methods

### 2.1. Search strategy

The protocol for this systematic review was prepared according to the Preferred Reporting Items for Systematic Reviews and Meta-Analyses (PRISMA) guidelines and prospectively registered with PROSPERO (CRD42021297136). The search strategy was developed with assistance from a clinical librarian. Search terms were related to iPSCs, ESCs, and DCCs and combined with keywords relevant for CIPN, using Boolean operators and truncation. Articles written in English and Dutch were considered for inclusion, whereas no restriction was placed on publication date. The literature search was performed on December 7, 2021, and updated on September 26, 2023, in MEDLINE (PubMed) and Embase (Ovid). The complete search is attached as Supplementary Material 1 (http://links.lww.com/PAIN/C11).

### 2.2. Eligibility criteria and study selection

Peer-reviewed experimental studies presenting original data and using relevant market available chemotherapeutic drugs were eligible for inclusion.

Considered articles had to describe development of CIPN models constructed from reprogrammed cells. Alternatively, studies that presented detailed information on outcome after treatment with chemotherapeutic agents were regarded as de facto model descriptions, and therefore also eligible for inclusion. Where applicable, intervention groups had to be compared with vehicle-treated or untreated neurons. Furthermore, studies had to mention outcome parameters relevant to model construction and validation, such as effects on neurite dynamics, cytotoxicity, and electrophysiological function.^11,27^ Studies that used only motor neuron cell culture systems were excluded.

Records were entered into Rayyan review software after removal of duplicates.^37^ Two researchers (P.S., K.H.) independently screened studies based on title and abstract and subsequently performed full-text screening. Thereafter, the reference lists of included studies were searched. Discrepancies were resolved between the 2 reviewers and, if needed, a third reviewer (N.W.).

### 2.3. Data extraction and presentation

Data concerning differentiated cell type and source, treatment (ie, agent, molar concentration, and treatment duration), and model validating assays was collected. Furthermore, to help guide investigators, we extrapolated the half-maximal inhibitory concentration (IC_50_) of chemotherapeutic drugs, regarding cytotoxicity and neurite dynamics, from text, graphs, or tables, where possible. Methodological differences in incubation time and molar concentration were deemed too large for statistical pooling and appropriate conduction of meta-analysis.

## 3. Results

### 3.1. Study selection

**Figure 1.**
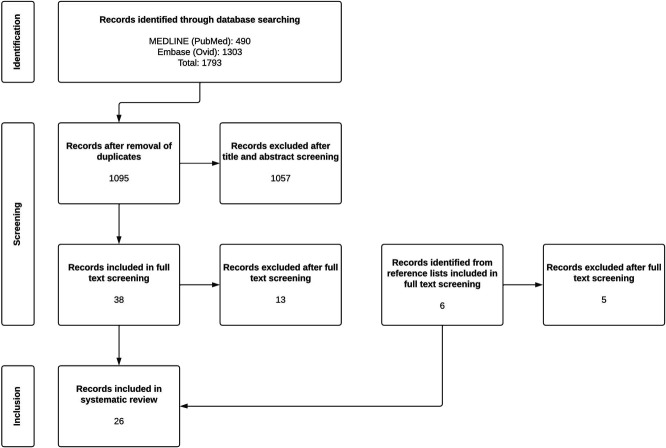
Flowchart of the literature search.

A total of 1095 unique records were found through database searches in MEDLINE (PubMed) and Embase (Ovid). Of these, 25 records were deemed appropriate for inclusion. Subsequent reference list searching of included articles identified 1 more eligible record. Thus, 26 articles were included in this systematic review. However, because 2 reports described the same experiments, these are hereafter consistently referenced as 1.^47,48^ The flow chart of the literature search is depicted in Figure 1. Table 1 summarizes the included studies.

**Table 1:** Overview of included studies.

Reference	Differentiated cell type*	Chemotherapeutic agent and dose	Treatment duration†	Model validated by
Komatsu et al., 2015	Cortical neurons of unspecified type (iCell)	Paclitaxel 1 nM—10 µM	48 hours	Immunocytochemistry Neurite dynamics (Calcein AM)
Lee et al., 2015	Peripheral (nociceptive) sensory neurons (from DCC)	Paclitaxel 1 nM—1 µM	48 hours	Immunocytochemistry Cell count (Hoechst) Neurite dynamics (Calcein)
Nakamura et al., 2015	Mixed population of GABAergic and glutamatergic cortical neurons (iCell)	Paclitaxel 1 µM Vincristine 100 pM Cisplatin 100 µM Oxaliplatin 100 µM Fluorouracil 100 µM	24 hours	Immunocytochemistry Cell count (propidium iodide) Live-cell imaging Intraneuronal transport (CM-Dil)
Wheeler et al., 2015	Mixed population of GABAergic and glutamatergic cortical neurons (iCell)	Paclitaxel 1 nM—100 µM Vincristine 1 nM—100 µM Cisplatin 1 nM—100 µM	24, 27, 48, 72 hours‡	Immunocytochemistry Neurite dynamics (Calcein AM) Live-cell imaging Neurite growth Cell viability analysis ATP Apoptosis induction analysis Caspase 3/7
Morrison et al., 2016	Mixed population of GABAergic and glutamatergic cortical neurons (iCell)	Paclitaxel 10 nM—100 µM Vincristine 10 nM—100 µM Cisplatin 10 nM—100 µM	72 hours	Immunocytochemistry Neurite dynamics (Calcein AM)
Hoelting et al., 2016	Peripheral (nociceptive) sensory neurons (from both iPSC and ESC)	Vincristine 80 pM—20 nM Cisplatin 1.56 µM—50 µM Bortezomib 640 pM—2 µM	24 hours	Immunocytochemistry Neurite dynamics (Calcein AM) Cell viability (Hoechst/Calcein AM)
Rana et al., 2017	Peripheral neurons (Peri.4U)	Paclitaxel 130 pM—10 µM Docetaxel 130 pM—10 µM Vincristine 10 pM—1 µM Cisplatin 1.3 nM—100 µM Oxaliplatin 1.3 nM—100 µM Carboplatin 1.3 nM—100 µM Thalidomide 1.3 nM—100 µM Lenalidomide 1.3 nM—100 µM Pomalidomide 1.3 nM—100 µM Bortezomib 1.3 nM—100 µM Ixabepilone 130 pM—10 µM	24 hours	Immunocytochemistry Neurite dynamics (β3-tubulin) Cell count (DAPI) Cell viability analysis ATP
Wing et al., 2017	Mixed population of GABAergic and glutamatergic cortical neurons (iCell) Peripheral neurons (Peri.4U)	Paclitaxel 10 pM—100 µM Docetaxel 10 pM—100 µM Nab-paclitaxel 10 pM—1 µM Vincristine 10 pM—100 µM Cisplatin 10 pM—100 µM Carboplatin 10 pM—100 µM Oxaliplatin 10 pM—100 µM Thalidomide 10 pM—100 µM Bortezomib 10 pM—100 µM Fluorouracil 10 pM—100 µM	4, 24, 48, 72 hours‡	Immunocytochemistry Neurite dynamics (Calcein AM) Cell viability analysis ATP Apoptosis induction analysis Caspase 3/7 Microelectrode array
Cohen et al., 2018	GABAergic cortical neurons (iCell) Glutamatergic cortical neurons (iCell)	Paclitaxel 100 nM—100 µM	24 hours	Immunocytochemistry Neurite dynamics (β3-tubulin) Cell count (DAPI) Cell viability analysis ATP
Snyder et al., 2018	GABAergic cortical neurons (iCell) Peripheral neurons (Peri.4U)	Paclitaxel 0.1 pM—10 µM Docetaxel 0.1 pM—50 µM Vincristine 0.1 pM—50 µM Vindesine 0.1 pM—50 µM Cisplatin 0.1 pM—50 µM Oxaliplatin 0.1 pM—50 µM Bortezomib 0.1 pM—50 µM Carfilzomib 0.1 pM—10 µM Eribulin 0.1 pM—50 µM	72 hours (measurements every 12 hours)	Immunocytochemistry Activated caspase 3/7 Phase contrast microscopy Neurite dynamics
Vojnits et al., 2019	Peripheral (nociceptive) sensory neurons (from DCC)	Paclitaxel 10 nM—10 µM Vincristine 10 nM—10 µM Cisplatin 10 nM—10 µM Bortezomib 10 nM—10 µM Etoposide 10 nM—10 µM	48 hours	Immunocytochemistry Neurite dynamics (Calcein AM) Cell count (Hoechst) Cell viability analysis Resazurin reduction assay
Chua et al., 2020	Peripheral (nociceptive) sensory neurons (differentiation was not confirmed by the authors)	Paclitaxel 1 µM	48 hours	Immunocytochemistry Neurite dynamics (β3-tubulin) Cell count (DAPI) Cell viability analysis ATP Apoptosis induction analysis Caspase 3/7
Chen et al., 2021	Peripheral sensory neurons	Vincristine 500 pM—5 nM	48 hours	Phase contrast microscopy Neurite dynamics
Diouf et al., 2021	Peripheral neurons (Peri.4U)	Vincristine 10 pM—10 µM	48, 72 hours	Immunocytochemistry Neurite dynamics (Calcein AM) Cell viability analysis ATP
Hrstka et al., 2021	Peripheral (nociceptive) sensory neurons	Bortezomib 10 nM—100 nM	24, 48 hours	Mitochondria transport assay Western blotting β3-Tubulin
Schinke et al., 2021§	Peripheral (nociceptive) sensory neurons	Paclitaxel 100 pM—10 µM Vincristine 100 pM—10 µM Cisplatin 1 nM—100 µM Bortezomib 100 pM—10 µM Fluorouracil 100 pM—10 µM	24, 72 hours (some assays were also performed 48 hours after 24 hours incubation)	Bright field microscopy Neuron morphology Live-cell imaging Neuron morphology (fluoresceine diacetate) Cell viability analysis MTT assay Protease activity
Wang et al., 2021	Peripheral (nociceptive) sensory neurons	Vincristine 1 nM—100 µM Cisplatin 1 nM—100 µM Pomalidomide 1 nM—100 µM Bortezomib 1 nM—100 µM Ixabepilone 1 nM—100 µM	24 hours	Immunocytochemistry Neurite dynamics (Calcein AM) Cell count (Hoechst) Cell viability analysis LDH release Apoptosis induction analysis Caspase 3/7
Xiong et al., 2021	Peripheral (nociceptive) sensory neurons	Paclitaxel 1 nM—100 µM Docetaxel 100 nM—1 µM Vincristine 10 nM—100 nM Bortezomib 100 nM—1 µM	24, 48, 72 hours‡	Immunocytochemistry Neurite dynamics (β3-tubulin) Cell count (DAPI) Cell viability analysis ATP Apoptosis induction analysis Caspase 3/7 Mitochondria transport and membrane potential assays
Cunningham et al., 2022	Peripheral sensory neurons (differentiation was not confirmed by the authors)	Paclitaxel 100 nM—1 µM	48 hours	Immunocytochemistry Neurite dynamics (β3-tubulin) Cell viability analysis ATP Whole-cell patch-clamping
Holzer et al., 2022a	Peripheral (nociceptive) sensory neurons Peripheral neurons (Peri.4U)	Paclitaxel 10 pM—100 nM Bortezomib 10 pM—100 nM Carfilzomib 10 pM—1 nM	24, 48, 72 hours‡	Immunocytochemistry Neurite dynamics (Calcein AM) Cell viability (Hoechst/Calcein AM) Calcium imaging
Holzer et al., 2022b	Peripheral (nociceptive) sensory neurons	Paclitaxel 500 pM—80 nM Cisplatin 600 nM—100 µM Oxaliplatin 500 nM—100 µM Bortezomib 1 nM—1 µM	1, 24 hours‡	Immunocytochemistry Neurite dynamics (Calcein AM) Cell viability (Hoechst/Calcein AM) Calcium imaging
Huehnchen et al., 2022	Peripheral (nociceptive) sensory neurons	Paclitaxel 100 pM—10 µM	24, 48, 72 hours‡	Live-cell imaging Axonal damage (Calcein) Cell viability analysis MTT assay Protease activity
Tsai et al., 2022	Peripheral (nociceptive) sensory neurons	Vincristine 0.48 nM—7.5 nM	48 hours	Immunocytochemistry Neurite dynamics (β3-tubulin) Neuron count (Peripherin)
Snavely et al., 2022	Peripheral (nociceptive) sensory neurons (differentiation was not confirmed by the authors)	Bortezomib 0.1 nM—15 nM	24, 48, 72 hours‡	Bright field microscopy Neuron morphology Live-cell imaging Neurite length
Mortensen et al., 2023	Peripheral (nociceptive) sensory neurons	Paclitaxel 0.1 µM—10 µM Fluorouracil 10 µM—1 mM	48 hours	Immunocytochemistry Neurite dynamics (neurofilament light chain) Single-molecule array (neurofilament light chain)

* Derived from iPSC, unless stated otherwise.

† Assays were performed after completion of the treatment, unless stated otherwise.

§ The original article by Schinke et al. and its separately published dataset were treated as one.

‡ Not all treatment durations were applied for all drugs/assays.

DCC, directly converted cell; iPSC, induced pluripotent stem cell; ECC, embryonic stem cell.

### 3.2. Aim of included studies

The included studies could be classified in 3 stages of complexity regarding their aim. The first group comprised of studies that performed elaborate neurotoxicity testing of different compounds but did not specifically list CIPN modelling as its aim (2 studies).^7,17^ The second group of studies aimed to model CIPN but stopped there (13 studies).^18,19,25,31,33,35,44,47,48,55,61,63,65,66^ Whereas the third group went beyond these efforts and subsequently applied its developed CIPN model to the study of pathophysiological mechanisms of CIPN (10 studies).^4,5,8,9,20–22,54,60,62^

### 3.3. Methodological characteristics of studies

#### 3.3.1. Reprogramming strategy and origin of neurons

The included publications reflect the advancements made in cellular reprogramming. As shown in Table 1, earlier studies mainly incorporated commercial cortical cell lines consisting of a mixed population of GABAergic and, to a lesser extent, glutamatergic neurons. Thereafter, models were constructed from pure populations of GABAergic neurons or commercial “peripheral” neurons. Two groups used both cortical and peripheral iPSC-derived cell lines and found higher sensitivity of peripheral cells to chemotherapy.^55,65^ Currently, most research groups incorporate peripheral nociceptive sensory neurons, primarily made using versions of the Chambers protocol, that aim to mimic nociceptive sensory DRG neurons.^3^ No publications were included that used co-culture of neurons and, for example, Schwann cells.

Three research articles describe the differentiation of either DCCs or ESCs, whereas all other articles used iPSCs as the starting material for experiments.^17,25,61^ Ten research groups used peripheral nociceptive sensory neurons differentiated from more than 1 donor or pluripotent stem cell line.^8,17,19–21,25,33,47,48,60,61^

#### 3.3.2. Antineoplastic treatment: drug, duration of treatment, and molar concentration

Chemotherapy-induced peripheral neuropathy models were constructed from a diverse group of antineoplastic agents (Table 2). Drug classes with the highest incidence of CIPN, that is, taxanes, vinca alkaloids, and platinum-based agents, were most represented. Bortezomib, a proteasome inhibitor, was also commonly studied. Numerous articles compared different drugs, but no combinations of drugs were tested in the same experimental setup.

**Table 2:** Overview of used chemotherapeutic agents.

Microtubule targeting agents			Platinum-based agents		
Taxanes			Cisplatin	11	17,18,31,35,44,47,48,55,61–63,65
Paclitaxel	18	5,7,8,18,19,21,22,25,31,33,35,44,47,48,55,61,63,65,66	Oxaliplatin	5	18,35,44,55,65
Nab-paclitaxel	1	65	Carboplatin	2	44,65
Docetaxel	4	44,55,65,66	Immunomodulatory agents		
Vinca alkaloids			Thalidomide	2	44,65
Vincristine	14	4,9,17,31,35,44,47,48,55,60–63,65,66	Lenalidomide	1	44
Vindesine	1	55	Pomalidomide	2	44,62
Epothilones			Miscellaneous		
Ixabepilone	2	44,62	Fluorouracil	4	32,35,47,48,65
Other			Etoposide	1	61
Eribulin	1	55			
Proteasome inhibitors					
Bortezomib	12	17–20, 44, 47, 48, 54, 55, 61, 62, 65, 66			
Carfilzomib	2	19,55			

Number of studies describing use of each drug.

Some model descriptions tested more than 1 drug.

The original article by Schinke et al. and the corresponding separately published dataset were treated as one, but both are referenced.

Although there seems to be no consensus on the preferred treatment duration, all authors found that treatment of 24 to 72 hours was sufficient for induction of CIPN. Ten studies tested multiple time points, whereas others treated cells for a single time point (either 24, 48, or 72 hours).^9,18–21,47,48,54,63,65,66^ Justification for the chosen treatment duration was mostly absent and no obvious patterns could be observed for specific drug(s) (classes). Assays were performed immediately after completion of treatment for all but 2 publications. Snyder et al. evaluated treatment effects every 12 hours for the 72-hour treatment, whereas Schinke et al. were the only to test neurotoxicity (48 hours) after treatment had finished.^47,48,55^ Apart from the latter study, none report data regarding long-term effects of chemotherapeutic treatment within these model systems.

Drug concentrations were highly variable, even between studies using the same agent (Table 1). Most publications tested a range of molar concentrations, whereas some report applying concentrations based on in vivo plasma concentrations. Below we outline the effects of the 4 (paclitaxel, vincristine, bortezomib, and cisplatin) most used drugs.

#### 3.3.3. Outcome parameters

A large number of different analyses were performed to assess the ability of chemotherapeutic agents to induce CIPN (Table 1). Most applied were (immunofluorescence) imaging techniques assessing morphological changes to neurites (used as a surrogate marker of microtubule disruption) and assays studying cell viability or toxicity parameters. Three articles investigated intraneuronal transport (of mitochondria).^20,35,66^ Only 4 research groups assessed the electrophysiological properties of neuropathic neurons, using microelectrode array, whole-cell patch-clamping, or calcium imaging.^8,18,19,65^

### 3.4. Drug effects in model systems

Paclitaxel, vincristine, bortezomib, and cisplatin stood out for their frequent usage (Table 2). Therefore, we extrapolated the IC_50_ of these drugs regarding neurite dynamics and cytotoxicity, in peripheral neurons.

**Figure 2.**
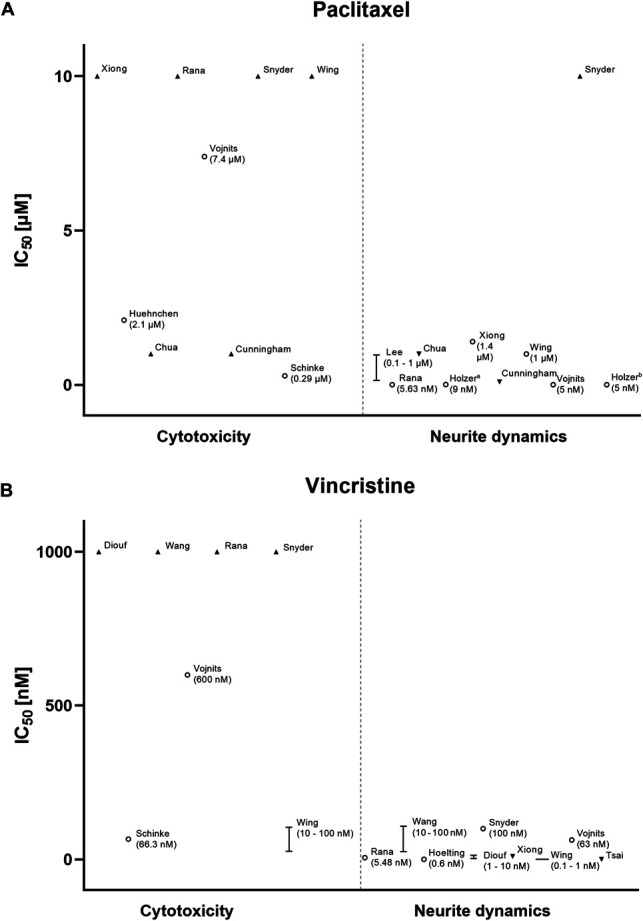
Peripheral neurons show differential sensitivity regarding cytotoxicity and neurite dynamics for paclitaxel and vincristine. Data points show the extrapolated half-maximal inhibitory concentrations (IC_50_) for paclitaxel (A) and vincristine (B). The results of the most mature, best-defined cell line and shortest treatment duration are depicted for studies that tested multiple strategies. Only assay-based results are shown for cytotoxicity. Importantly, IC_50_s of chemotherapeutic agents might be dependent on factors (ie, specific assay and cell line, treatment duration, and cell age) that could not, or only partially, be accounted for in this figure. Data were extrapolated from text, graphs, and tables.

**Figure 3.**
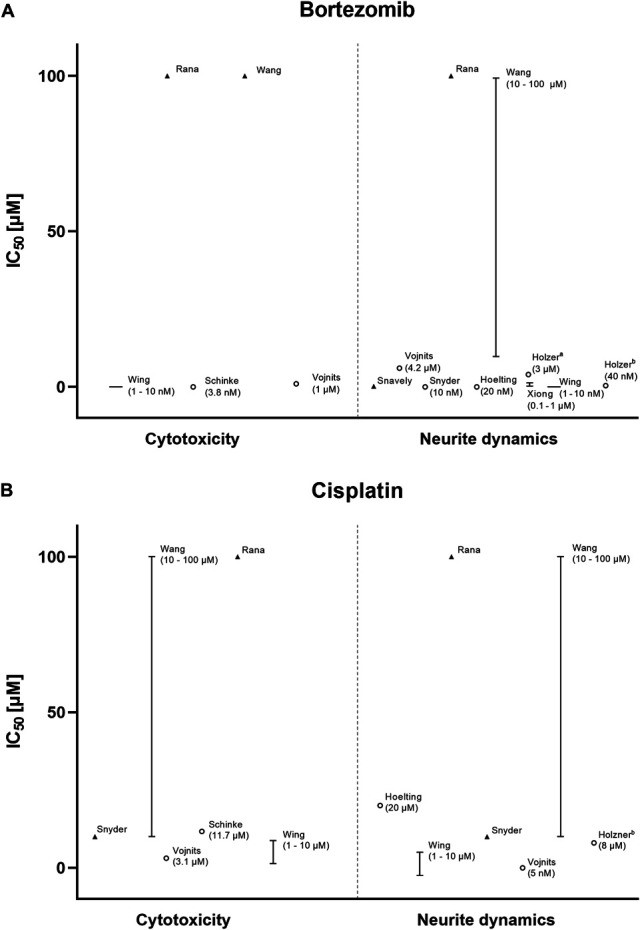
Bortezomib and cisplatin have an overlapping effect on cytotoxicity and neurite dynamics in peripheral neurons. Data points show the extrapolated half-maximal inhibitory concentrations (IC_50_) for bortezomib (A) and cisplatin (B). The results of the most mature, best-defined cell line and shortest treatment duration are depicted for studies that tested multiple strategies. Only assay-based results are shown for cytotoxicity. Importantly, IC_50_s of chemotherapeutic agents might be dependent on factors (ie, specific assay and cell line, treatment duration, and cell age) that could not, or only partially, be accounted for in this figure. Data were extrapolated from text, graphs, and tables.

Paclitaxel, a microtubule-stabilizing drug that prevents mitosis in cancer cells, was found to display a large difference in sensitivity for cytotoxicity and neurite dynamics (Fig. 2A). A similar profile was found for vincristine, another microtubule-targeting agent (Fig. 2B). Bortezomib, a drug that inhibits proteasomes resulting in ubiquitinated protein accumulation, showed an overlap between cytotoxicity and neurite impairment (Fig. 3A). This was also seen for platinum-based cisplatin (Fig. 3B).

## 4. Discussion

This systematic review includes 26 peer-reviewed studies using reprogrammed human cells to model CIPN. Reflecting the progress in human cellular reprogramming, we observed a shift in used cell type from cortical neurons to nociceptive DRG neurons. Models were almost exclusively validated by assessment of the acute effects of chemotherapeutic agents. Drugs with the highest incidence of CIPN were most represented within the model systems. Emphasizing the power of cellular reprogramming, part of the included studies were already aimed at elucidating the pathophysiological mechanisms of CIPN.

Different models of CIPN have been used over the last decennia. These include neuronal-like cell lines (eg, rat PC12 and neuroblastoma-derived SH-SY5Y), primary rodent DRG explants or dissociated cell cultures, and numerous animal (typically mice) in vivo models.^10,14,27^ Although these model systems have helped improve knowledge on the pathophysiological mechanisms of CIPN, no substantial gains have been made toward improvements in prevention and treatment of CIPN.^2,11^ This is often speculated to be due, in part, to the methodological and translational limitations of the current models. For example, the impaired reproducibility of primary cultures, genetic instability, and altered physiology of immortalized cell lines and unexpected variation in sensitivity to chemotherapy between different species and strains of animals all hinder clinical translation.^12,27,30,38,41^ Yet perhaps most importantly, inherent translational problems exist with results from animal studies because of genetic differences in the nociceptive system between humans and other species.^39,46,49,51,59^ Combined these issues indicate that (current) laboratory results interpreted as in vitro surrogate markers of clinical features of CIPN might not fully translate to human in vivo symptoms, such as neuropathic pain. Thus, an improved complementary human in vitro model of CIPN is needed.

Developments in human cellular reprogramming now allow for the creation of human nociceptive DRG neurons from unrelated cell types, such as umbilical cord blood cells.^3^ Thereby, the technology enables a method of disease modelling that overcomes many of the aforementioned problems.^24,29,52^ Specifically, strengths of human cellular reprogramming include its ability to produce theoretically unlimited quantities of otherwise inaccessible human cells, while concurrently providing more translatable results by enabling application of previously unattainable humanized in vitro models.^53^ Cellular reprogramming also offers the option of constructing patient-specific models using patients' cells, and important for CIPN modelling, acquired nociceptive DRG neurons correctly recapitulate chemotherapeutic substance specificity as observed clinically (eg, demonstrated by Schinke et al.).^24,47,48,53^ Yet the technique also has limitations. For example, terminal differentiation after reprogramming is currently still a relatively expensive, time-consuming, and labor-intensive process.^53^ Further, reprogramming could induce more variability than seen in primary DRG cultures, whereas the maturation status of obtained cells is another factor to consider.^50^ Incomplete differentiation could result in an impaired epigenetic status, whereas mature expression patterns of protein markers and correct electrophysiological functionality might require extended periods of culture.^42,45,67^

The studies included in this systematic review were the first to take advantage of this technique for CIPN. However, obvious from these reports is that modelling CIPN using reprogrammed cells is still in its infancy. Substantial heterogeneity between studies was visible in the way researchers applied chemotherapy and reported their findings. For example, paclitaxel concentrations ranged from 0.1 pM to 100 μM, although reasoning for the chosen concentration(s) was largely missing. Description of the reprogrammed cells also differed much between studies. Where some, such as Schinke et al., report the results of exhaustive morphological and functional characterization, others did not check the results of differentiation (Table 1).^47,48^ In this regard, the efforts of the authors who used multiple pluripotent cell lines should also be credited.^8,17,19–21,25,33,47,48,60,61^ Furthermore, most of the reports validated treatment outcome solely by assessing the acute effects on neurite dynamics and cytotoxicity parameters, even though these may not be the only appropriate endpoint for antineoplastic drugs and should ideally be complemented by functional assays. This is exemplified by the fact that only 4 studies inspected the electrophysiological outcome of chemotherapeutic treatment.^8,18,19,65^ Additionally, sought after treatment effects were not prospectively formulated. Therefore, it is uncertain whether all the included studies were successful in recapitulating CIPN to its full extent.

### 4.1. Recommendations for future chemotherapy-induced peripheral neuropathy model development

Reliability and standardization of preclinical models, however, is crucial for their successful use in (pharmacological) research.^11,27^ Based on the results of this systematic review and expert opinion, we propose a number of suggestions to improve the clinical relevance and appropriateness of human cellular reprogramming-derived CIPN models.

First, we recommend the use of well-defined differentiated cell types and subsequent detailed description of differentiation protocols in publications. This should include reporting of a core set of cellular markers (eg, β3-tubulin, BRN3A, and TRPV1 in case of nociceptive DRG neurons). Cells may be of commercial origin; however, these are often not well characterized. Second, considering the impact of genetics on CIPN susceptibility and reprogramming-induced variability in iPSC cell lines, we suggest to use cells from multiple donors within the same project.^6,23,57^ Third, as the human DRG contains many different cell types, co-culture of neurons with, for example, Schwann cells or macrophages might improve clinical translatability and should be considered.^23,26,34,36,40,69^ Also the application of three-dimensional organoids may be of interest, however, their use in high-throughput (pharmacological) research is still limited by impracticalities, such as phenotypic variability and inaccessibility for microscopy assays.^23,69^ Fourth, future models should be validated using multiple methods that reflect the ongoing progress in pathophysiological knowledge. Importantly, this should include electrophysiological assays (eg, microelectrode array, whole-cell patch-clamping or calcium imaging) that can assess neuronal signal transduction as a surrogate maker of neuropathic pain.^1,56^ Fifth, because neuropathic effects may worsen after discontinuation of treatment with some antineoplastic agents, we advise to assess outcome also at later time points to factor in the phenomenon of coasting.^15,43^ Finally, to enhance transparency and facilitate the comparison of research results between laboratories, researchers should make their datasets accessible and include data availability statements in their manuscripts.

### 4.2. Strengths and limitations

This systematic review adhered to a prospectively registered protocol to ensure transparency and increase reproducibility. Furthermore, we followed the PRISMA guidelines and performed independent record screening and data extraction. A clinical librarian was consulted to help develop the search strategy and maximize relevant search results. Combined these methodological strengths explain why this study was able to extract twice as many high-quality reports than another recently published systematic review.^32^

The main limitation of this study relates to the heterogeneity of the individual reports, in both model construction and reporting. Conduction of meta-analysis was deemed inappropriate and, thus, only minimal guidance can be offered regarding drug concentrations. To combat this, we extracted and plotted the IC_50_s of the most frequently used drugs; however, for some studies we were only able to provide estimates. Differing quality of reporting also made it difficult to properly assess the internal validity of studies; yet, they seem to be largely in agreement with each other regarding cytotoxicity and neurite outgrowth endpoints (Figs. 2 and 3).

## 5. Conclusions

This systematic review describes the first studies that used human cellular reprogramming techniques to model CIPN. Considerable differences in methodology were found, thus limiting comparability and translatability of studies. Therefore, a number of recommendations are provided for prospective researchers interested in modeling CIPN. Ultimately, these humanized models hold the potential to enlarge pathophysiological knowledge and aid in the discovery of novel intervention strategies.

## Conflict of interest statement

The authors have no conflicts of interest to declare.

## Appendix A. Supplemental digital content

Supplemental digital content associated with this article can be found online at http://links.lww.com/PAIN/C11.

## Supplementary Material

SUPPLEMENTARY MATERIAL
