# Supplementary material for: Chemotherapy-induced peripheral neuropathy models constructed from human induced pluripotent stem cells and directly converted cells: a systematic review
Source: Pain. 2024 Feb 21;165(9):1914–25. doi: 10.1097/j.pain.0000000000003193 (PMC11331829; doi:10.1097/j.pain.0000000000003193)
Supplement: SUPPLEMENTARY MATERIAL [file jop-165-1914-s001.pdf]

# Chemotherapy-Induced Peripheral Neuropathy Models Constructed from Human Induced Pluripotent Stem Cells and Directly Converted Cells: A Systematic Review

Pascal S.H. Smulders[1], Kim Heikamp[1], Jeroen Hermanides[1], Markus W. Hollmann[1], Werner ten Hoope[1,2], Nina C. Weber[1]

**From the:**

1: Amsterdam UMC location University of Amsterdam, Laboratory for Experimental Intensive Care and Anesthesiology (L.E.I.C.A.), Department of Anesthesiology, Amsterdam, The Netherlands.

2: Rijnstate Hospital, Department of Anesthesiology, Arnhem, The Netherlands.

**Corresponding author:**

Markus W. Hollmann, Amsterdam UMC location University of Amsterdam, Department of Anesthesiology, Amsterdam, The Netherlands. Phone number: +31 (0)20 7323932. E-mail address: m.w.hollmann@amsterdamumc.nl.

**Supplementary Material**

## Supplementary Material 1 – Search strategy

Clinical librarian: Faridi Jamaludin, Amsterdam UMC, Medical Library AMC

Date of search: December 7 2021, updated on September 26 2023.

Databases: MEDLINE (PubMed) and Embase (Ovid).

**Search strategy MEDLINE (PubMed):**

("Human Embryonic Stem Cells"[mesh] OR "Induced Pluripotent Stem Cells"[mesh] OR human embryonic stem cell*[tiab] OR induced pluripotent stem cell*[tiab] OR iPS cell* [tiab] OR iPSC*[tiab] OR direct conver*[tiab] OR direct reprogram*[tiab])

AND

("Peripheral Nervous System Diseases/chemically induced"[MeSH] OR "Polyneuropathies/chemically induced"[Mesh] OR induced peripheral neuropath*[tiab] OR induced neuropath*[tiab] OR CIPN[tiab] OR induced neurotoxicit*[tiab] OR neurotox*[tiab] OR neuronal toxicit*[tiab] OR sensory peripheral neuropath*[tiab])

**Search strategy Embase Classic and Embase (Ovid):**

| # | Entries |
|---|---|
| 1 | human embryonic stem cell/ or induced pluripotent stem cell/ |
| 2 | (human embryonic stem cell* or induced pluripotent stem cell* or iPS cell* or iPSC* or direct conver* or direct reprogram*).ti,ab,kf. |
| 3 | 1 or 2 |
| 4 | chemotherapy-induced peripheral neuropathy/ or peripheral neuropathy/ or neurotoxicity/ |
| 5 | (induced peripheral neuropath* or induced neuropath* or CIPN or induced neurotoxicit* or neurotox* or neuronal toxicit* or sensory peripheral neuropath*).ti,ab,kf. |
| 6 | 4 or 5 |
| 7 | 3 and 6 |
